# Barriers affecting medication adherence to dienogest among patients with ovarian endometriosis: a qualitative study

**DOI:** 10.3389/fmed.2026.1749740

**Published:** 2026-01-30

**Authors:** Yihui Duan, Chaonan Wang, Yuyi Luo, Shuming Guo

**Affiliations:** 1 School of Nursing, Shanxi Medical University, Taiyuan, China; 2 Hospital Administration Office, Linfen Central Hospital, Linfen, China

**Keywords:** ovarian endometriosis, dienogest, medication adherence, barriers, qualitative interview, nursing

## Abstract

**Objective:**

To explore barriers affecting medication adherence (e.g., missed doses, forgotten doses, or discontinuation) among patients with Ovarian Endometriosis (OE) using the Capability, Opportunity, Motivation – Behaviour (COM-B) behavioural change model as a theoretical framework and propose targeted improvement measures.

**Methods:**

A qualitative research approach was used. From March to May 2025, interviews were conducted with 15 patients diagnosed with Ovarian Endometriosis and taking dienogest for over six months at a Grade A tertiary hospital in Linfen City. Data were analysed using directed content analysis.

**Results:**

Three themes were identified: lack of capability, lack of opportunity, and insufficient patient motivation.

**Conclusion:**

This study employed semi-structured interviews combined with the COM-B behavioural change model to systematically analyse barriers affecting dienogest medication adherence among patients with Ovarian Endometriosis. It deeply explored their experiences and perceptions of medication use, providing a reference for developing subsequent intervention strategies to enhance patient medication adherence.

## 1 Introduction

In recent years, with increased disease awareness and advances in diagnostic technology, the number of confirmed cases of Ovarian Endometriosis (OE) has been steadily rising worldwide, necessitating optimized management strategies. Ovarian Endometriosis (OE) is a common oestrogen-dependent chronic inflammatory disease among women of reproductive age. As the most prevalent form of endometriosis (EMS) (1), it accounts for 30–40% of all cases (2, 3).

Current treatment approaches for endometriosis primarily involve medical management and surgical interventions. The fundamental nature of disease necessitates continuous medical therapy to counteract its pathophysiological processes (3). Patients meeting surgical criteria still require long-term medical management postoperatively to prevent recurrence (4, 5).

Dienogest, recommended as a first-line agent in domestic and international guidelines, significantly reduces recurrence risk through potent progesterone receptor agonism (6, 7). However, its strict requirement for consistent daily dosing at the same time without interruption is efficacy-dependent, necessitating sustained plasma concentrations within a 24-h window. Irregular administration may cause fluctuating oestrogen levels, leading to adverse outcomes such as breakthrough bleeding and increased pain (8). Clinical evidence indicates that patients with high medication adherence exhibit significantly lower recurrence risks than those with low adherence (9), and sustained use contributes to improved pregnancy success rates (10).

Previous studies on medication adherence among patients with OE have predominantly employed quantitative methodologies, focusing primarily on factors such as adverse effects, safety, and efficacy (6, 11, 12). These studies lack an in-depth exploration of the root causes of the disorder and patients’ lived experiences, as well as an examination of the interplay between psychological, behavioural, and social contexts.

**Figure 1:**
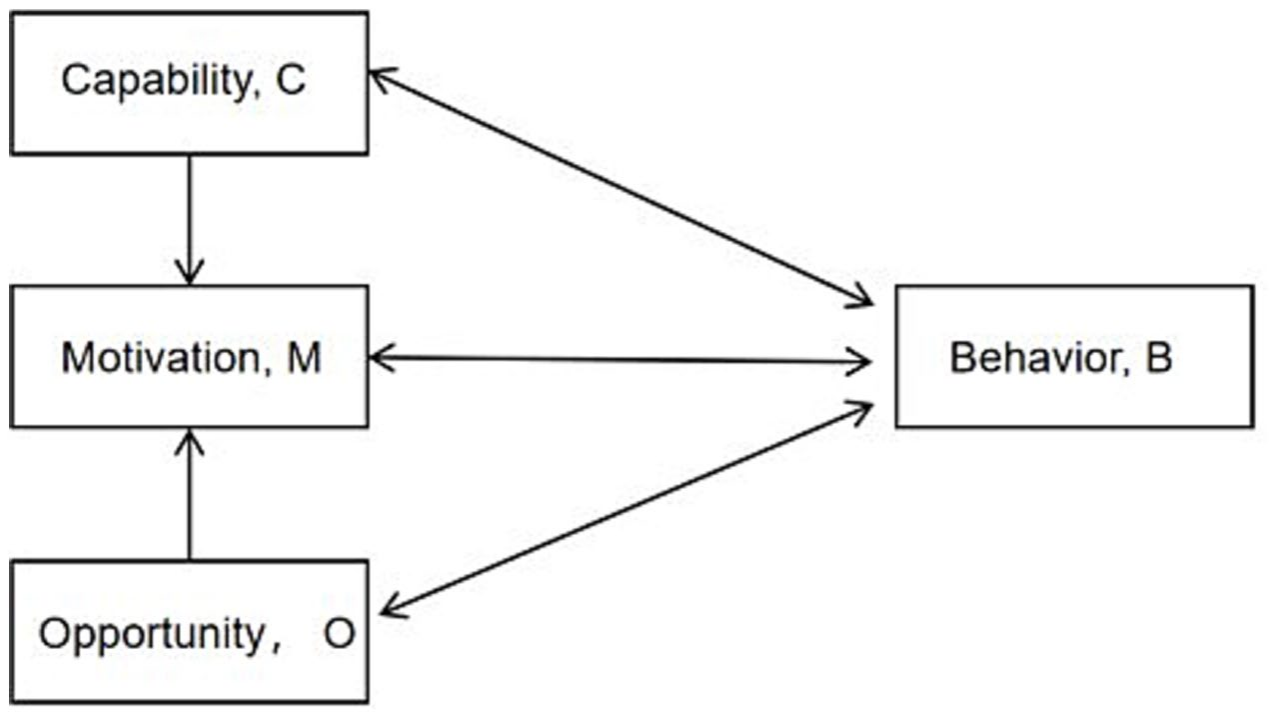
COM-B system—a framework for understanding behaviour.

The COM-B behavioural change model, proposed by Michie et al. (13), posits that behavioural change (B) is influenced by capacity (C), opportunity (O), and motivation (M). Capacity and opportunity may directly influence behaviour or indirectly affect it through motivation (Figure 1). This model provides a comprehensive and systematic understanding of factors that promote or hinder individual behavioural change. It has been applied by researchers worldwide across various diseases with favourable outcomes (14).

Therefore, this study employs the COM-B behavioural change model as its theoretical framework, grounded in the decisive role of dienogest adherence in disease outcomes. Utilising qualitative research methods and adopting a patient-centred perspective, this study aims to explore the adherence barriers experienced by patients with OE during dienogest treatment. This investigation seeks to understand the patient’s lived experiences and perceptions throughout medication use, examining how capability, opportunity, and motivation interact to influence adherence behaviour. This approach addresses the limitations of previous quantitative research, providing a structured foundation for developing targeted, multilevel, evidence-based nursing interventions to optimise clinical practice.

## 2 Design

Qualitative research provides deeper insights into patients’ experiences with dienogest. Content analysis, one of the most widely used techniques in qualitative research—particularly in nursing—aims to generate knowledge and understanding of the phenomena under investigation. As this study sought to understand the experiential factors contributing to medication adherence barriers among patients with OE, a qualitative approach employing content analysis was adopted (15).

## 3 Research methods

### 3.1 Establishment of the research team

The team comprised nine members: one gynaecology head nurse, five gynaecology nurses (two senior nurses and three registered nurses), and three nursing postgraduate students, including the researcher. All participants received qualitative research training and were familiar with the study content and requirements. The interviews were primarily conducted by the first author (Duan), with the remaining members (Wang and Luo) undertaking the coding and analysis of the data.

### 3.2 Ethical approval and compliance

This study was approved by the Ethics Committee of Linfen Central Hospital (approval no.: LY2025-14-01). This study strictly adhered to the ethical principles of the Declaration of Helsinki and was conducted in accordance with the Consolidated Criteria for Reporting Qualitative Research (COREQ). All participants signed informed consent forms.

### 3.3 Research subjects

Using purposive sampling, patients with ovarian endometriosis receiving dienogest treatment at Linfen Central Hospital were selected between March and May 2025. The inclusion criteria were as follows: (1) clinically diagnosed with ovarian endometriosis via triad examination, colour Doppler ultrasound, or laparoscopic surgery with pathological confirmation; (2) currently undergoing dienogest therapy; (3) duration of medication ≥ 6 months; (4) age ≥ 18 years; and (5) informed consent and voluntary participation in the study. The exclusion criteria were as follows: (1) lack of basic communication and comprehension abilities, and (2) concurrent severe mental illness or cognitive impairment. Sample size was determined by data saturation, defined as the point at which no new themes emerged from the interview data.

### 3.4 Development of interview guide

Based on the research objectives, relevant domestic and international literature was reviewed. Combining the COM-B theoretical framework, the research team experts collaboratively discussed and drafted a preliminary interview outline. Prior to the formal interviews, two patients underwent pilot interviews. The team further discussed, refined, and finalized the interview questions. (1) Has your doctor explained the mechanism of action of dienogest and why it must be taken long-term? (2) Are you clear about the rules for taking a missed dose? (3) Do you understand how to manage common side effects? (4) What measures do you take after missing a dose? (5) Does the cost of dienogest cause financial constraint? How much is covered by insurance? (6) Does the follow-up appointment process affect your confidence in continuing medication use? (7) Are your family members aware of your medication needs? Do they help remind or monitor your medication use? (8) Does your work/study environment facilitate taking medication on time? (9) What factors motivated you to adhere to medication? What emotions arise when you miss a dose? Do these emotions affect subsequent medication adherence? (10) Please describe a typical scenario where you missed a dose. The detailed correspondence between the interview outline and the COM-B model is provided in Supplementary Table S1.

### 3.5 Interview preparation

A quiet, private space and test recording equipment were selected beforehand. One postgraduate student (Wang) assisted during the interview.

### 3.6 Data collection and quality control

This study employed semi-structured interviews. The sequence of questions may be adjusted spontaneously during the interview, and the scope may be broadened to accommodate emerging themes. (1) Researchers will conduct targeted interviews with each patient using pre-established questions that adhere to the principles of non-directive and non-suggestive questioning. (2) Prior to the interviews, researchers introduced themselves and fully explained the study’s objectives, significance, and confidentiality principles. With respect to participants’ wishes, informed consent was obtained with their understanding and cooperation, and patients were informed of their right to withdraw at any point during the interview. (3) Interviews were conducted in a quiet, tidy reception room free from foot traffic. (4) The entire interview process was audio-recorded. Non-verbal behaviours of research participants were observed during the interview. When encountering ambiguous expressions, techniques such as repetition and probing were employed to encourage expression, ensuring the authenticity and reliability of recorded content. (5) Interviewers flexibly adjusted the sequence of questions based on the interview outline and practical needs to ensure the continuity and completeness of the interview data. Each interview lasted 30–60 min.

### 3.7 Data analysis

Following the interviews, two trained researchers (Duan and Luo) transcribed and organised the audio recordings and notes within 24 h. The transcribed texts were returned to the interviewees for verification. Following confirmation, the two researchers independently analysed and cross-checked the data to ensure accuracy and authenticity. Both researchers utilised NVivo software (version 12.0) to independently code, manage, and analyse all data, including non-verbal information. Disagreements were resolved through group discussions to reach a consensus, thereby enhancing the reliability of the findings. Data analysis employed the directed content analysis method, combined with the COM-B behaviour change model, to extract and elaborate themes.

## 4 Result

### 4.1 Patient basic information

This study ultimately included 15 patients, numbered P1–P15 (Table 1).

**Table 1:** General information of patients (*n* = 15).

Patient ID	Age	Educational attainment	Marital and fertility status	Occupation	Duration of medication use (months)	VAS score	Living with family	Method of medical payment
P1	23	Undergraduate	Single, no children	Clerical staff	6	3	No	Employee medical insurance
P2	26	Master’s Degree	Unmarried, childless	Student	8	2	Yes	Resident medical insurance
P3	42	Secondary School	Married with children	Freelancer	7	4	Yes	Resident medical insurance
P4	35	Junior Secondary School	Married with children	Freelancer	8	5	Yes	Self-funded
P5	30	Secondary School	Married with children	Freelancer	7	4	Yes	Self-funded
P6	35	Junior High School	Married with children	Freelancer	6	6	Yes	Resident medical insurance
P7	33	Secondary School	Married with children	Freelancer	9	4	Yes	Resident medical insurance
P8	37	Secondary School	Married, no children	Freelancer	8	5	Yes	Resident medical insurance
P9	33	Junior Secondary School	Married with children	Unemployed	6	8	No	
P10	33	Undergraduate	Married with children	Civil servant	6	2	Yes	Employee medical insurance
P11	43	Secondary Education	Married with children	Freelancer	8	4	Yes	Resident medical insurance
P12	28	Undergraduate	Unmarried and childless	Freelancer	6	3	Yes	Resident medical insurance
P13	37	Junior Secondary School	Married with children	Freelancer	13	5	Yes	Resident medical insurance
P14	30	Bachelor’s Degree	Married with children	Freelancer	7	5	Yes	Resident medical insurance
P15	34	Master’s Degree	Married with children	Clerical Staff	9	1	Yes	Employee medical insurance

VAS score, full name visual analogue scale, is a commonly used tool for quantifying pain assessment. 0 points: indicates the patient experiences no pain whatsoever. 1–3 points: mild pain, typically tolerable and not affecting daily life. 4–6 points: moderate pain, causing noticeable discomfort that may affect sleep and daily activities. 7–9 points: severe pain, intense and difficult to tolerate, significantly impacting sleep and daily life. 10 points: excruciating pain, typically intolerable and requiring urgent intervention.

Data analysis identified 3 themes and 10 subthemes: barriers in the capability dimension (Core Pharmacological Misunderstandings, Cognitive Biases Regarding the Disease, Lack of clarity on missed dose management rules); barriers in the opportunity dimension (financial burden and inadequate resource allocation, insufficient healthcare system support and follow-up, dysfunctional social support networks, severe stigma and shame associated with illness); and motivation-related barriers (concerns about medication side effects and long-term safety, delayed perception of efficacy versus disease threat, low patient self-efficacy).

#### 4.1.1 Theme 1: barriers in the capability dimension

##### 4.1.1.1 Core pharmacological misunderstandings

The majority of respondents demonstrated a limited understanding of the mechanism of action of dienogest. They failed to recognise that irregular medication intake disrupts the maintenance of stable, effective drug concentrations in the body, thereby reducing efficacy and potentially inducing breakthrough bleeding or symptom recurrence. Their perception of the medication remained confined to “treating cysts” or “pain relief.” Two patients (P5 and P14) pointed out:

*"The doctor only said it controls the cyst from growing larger; I don't quite understand how it works". "It feels much like painkillers – take it when it hurts, but can you stop once the pain subsides?"*

Furthermore, some patients inadequately grasped the distinctions between “asymptomatic ≠ cured” and “high recurrence risk after discontinuation,” harbouring the misconception that “smaller cysts/no pain = full recovery. This led to self-initiated dose reduction or discontinuation. Two patients (P6 and P8) pointed out:

*"Last month's check-up showed the cyst had shrunk, so I thought I was nearly cured. I forgot to take it for a few days, and now it's painful again this month. My cyst is now just 0.1mm. I reckon I can skip the pills for now and start again when it grows bigger, right?"*

Some patients lack a thorough understanding of the necessity of “strictly adhering to scheduled medication times and doses.” A patient (P9) said:

*"After a while, I stopped paying much attention. I just thought, 'As long as I take it eventually, missing a dose here and there won't hurt.'"*

##### 4.1.1.2 Cognitive biases regarding the disease

Interviews revealed significant misconceptions regarding the pathophysiology of the condition, with a persistent lack of clarity about the core diagnosis, even among patients who had undergone drug therapy for over six months. Furthermore, patients underestimated the necessity of long-term disease management, failing to fully grasp the critical role of sustained medication in controlling disease progression, preserving reproductive function, and preventing complications. They harboured doubts about the safety of prolonged medication, expressed anxiety about unknown risks, and tended to seek curative surgery. These cognitive biases not only influenced their choice of appropriate disease control behaviours but also undermined their resolve to actively manage the condition. Two patients (P8 and P11) pointed out:

*"How long must I take this? Could it affect future pregnancies? Even the doctor couldn't say. Might constant medication damage my liver or kidneys? Better to just have surgery and be done with it".*

Misinformation circulating among fellow patients further influences medication decisions, potentially setting negative precedents that erode confidence and motivation. P6 mentioned:

*"My fellow patient told me her abdominal pain intensified after ten days of medication—utterly unmanageable, with vomiting, diarrhoea, dizziness and dry mouth. Terrified, I hesitated to start. After two days, she stopped on her own".*

##### 4.1.1.3 Lack of clarity on missed dose management rules

Interviews revealed that over half of the patients could not clearly recall the 12-h missed-dose rule. Three patients (P6 and P7) stated:

*"I don't know if I should take it after 12 hours? Would taking two tablets cause problems? I usually take my medication at 9 pm. One day I forgot, only remembering the next evening. But by then the doctors had finished work. I didn't know whether to take it or who to ask".*

Patients lack knowledge on managing common side effects (e.g., spotting), leading to panic and self-discontinuation of medication. P3 stated:

*"Once I bled for over ten days. I was terrified and stopped the medication for several days before daring to ask the doctor".*

#### 4.1.2 Theme 2: barriers in the opportunity dimension

##### 4.1.2.1 Financial burden and inadequate resource allocation

Interviews revealed that the cost of dienogest places a significant strain on most patients, particularly those without stable income or with low medical insurance reimbursement rates. Three patients (P1, P2, and P3) said:

*"A single box of medicine accounts for one-fifth of my salary. I'm only 33, with decades before menopause, and no employee medical insurance. The pressure of continuously taking imported medication is overwhelming. As a student with no income, having to allocate money from my living expenses each month for medication is truly stressful. My basic medical insurance only covers three cycles. After that, I must purchase it at full price – over 500 yuan monthly. By my calculations, this medication costs more than gold".*

Additionally, many patients reported inadequate primary healthcare resources, highlighting urgent societal attention and systemic improvements. For instance, some cite medication access difficulties (e.g., hospital pharmacy shortages, regular trips to major hospitals). Two patients (P8 and P13) said:

*"My family lives in the county, and the county hospital cannot prescribe this medication. Every month, I must drive over an hour into the city to obtain it, and they only dispense one box at a time. On several occasions, I only remembered to get a new supply when my medication was nearly depleted, meaning I started the next month's treatment late. I genuinely cannot afford the constant travelling back and forth. Sometimes, between collecting medication and undergoing tests, I cannot return home within a single day. The round-trip travel costs, accommodation, and various examinations all add up to significant expenses".*

##### 4.1.2.2 Insufficient healthcare system support and follow-up

Two patients (P10 and P11) indicated that the cumbersome nature of the follow-up procedures further eroded treatment confidence, with some patients reporting that the complexities of scheduling appointments and lengthy waiting times undermined their commitment to ongoing care.

*"Spending the entire morning queuing for an appointment, only to receive a two-minute consultation. Feeling undervalued gradually makes one reluctant to return to hospital—it's simply too troublesome. Requiring monthly prescriptions, even with scheduled appointments, does not guarantee access to the same consultant overseeing my condition. Variations in treatment approaches and medication recommendations between doctors necessitate repeatedly explaining my medical history".*

Two patients (P11 and P15) raised concerns about follow-up care, revealing issues such as excessively long intervals between appointments or irregular scheduling. This prevents the timely detection and management of side effects, addresses patient enquiries, assesses medication adherence, and reinforces health education. Utilisation of structured medication reminder services (via APPs) was low and ineffective, with extremely low participation from healthcare providers. Only a small number of patients received reminders, which were easily overlooked.

*"I have a dedicated app on my phone that reminds me to take my medication, but it gets drowned out by the sheer volume of notifications. Apart from registering for appointments at the hospital, I've never received any medication reminders from hospital staff".*

##### 4.1.2.3 Dysfunctional social support networks

In chronic disease management, positive behavioural reinforcement mechanisms and family support systems are two critical factors influencing patient self-management outcomes (16). Ideally, timely affirmation and appropriate rewards can help patients establish stable health behaviour patterns through positive reinforcement. However, in practice, such reinforcement mechanisms are often absent. This leads to a lack of sustained behavioural feedback and motivation for patients, not only hindering the development of healthy habits but also causing persistent psychological energy depletion. A patient (P2) said:

*"I've been taking medication for a year now, yet scarcely anyone remarked on how commendable it is that I've persevered this long. At follow-up appointments, the doctor merely said 'Keep it up', without any specific praise or encouragement. While I understand this is my own responsibility and not others', this psychological fatigue may prove more difficult to bear than the illness itself".*

Concurrently, the dynamic shifts in familial support significantly impact patients’ self-management capabilities. Interviews revealed that during the initial diagnosis phase, patients typically received ample emotional care and practical assistance from family members. However, as the disease progresses and stabilises, this support tends to naturally diminish over time. As the patient (P6) pointed out:

*"At first, my husband would set alarms with me, saying he'd supervise my daily medication. But after a while, he grew a bit weary of it".*

##### 4.1.2.4 Severe stigma and shame associated with illness

‘Stigma’ refers to the shame patients internalise due to their illness, reflecting a psychological stress response (17). Societal taboos surrounding gynaecological conditions and menstrual issues may deter patients from seeking support or openly managing their medication. This socio-cultural environment exacerbates patients’ psychological burden, substantially impacting treatment outcomes. Two patients (P6 and P15) indicated:

*"Every time I meet friends for a meal, I have to sneakily take my medicine, terrified someone will see. Just pulling out the pillbox triggers a barrage of questions: “Are you sick? What's wrong with you?” This interrogation makes me feel incredibly embarrassed. It feels shameful to have this disease at such a young age".*

#### 4.1.3 Theme 3: motivation-related barriers

##### 4.1.3.1 Concerns about medication side effects and long-term safety

Interviews revealed that patients’ fear of hormone-related side effects (reduced metabolism, mood swings, and thrombosis risk) constituted a core barrier. Some patients were “terrified at the mere mention of hormones,” harbouring intense apprehension about hormonal medications, particularly concerning adverse effects on fertility, which undermined their willingness to adhere in the long term. Two patients (P4 and P13) said:

*"Sometimes I find the side effects of hormones more frightening than the pain itself – weight gain, depression, hair loss. I dread becoming like that. I'd rather endure the pain than take the medication. I've heard that long-term use can cause blood clots, which leaves me feeling quite unsettled".*

##### 4.1.3.2 Delayed perception of efficacy versus disease threat

To effectively manage their condition and improve their quality of life, patients with OE must adhere to a series of long-term and demanding control tasks. However, these behaviours often require significant effort and patience from patients, with noticeable results taking time to manifest. Some patients may experience minimal symptom improvement during the initial stages of treatment, leading to a lack of recognition of the importance of consistent treatment. A patient (P2) said:

*"I've been taking the medication for eight months now, but the cyst is still just as large—it's only shrunk by 3mm. I feel the effect is negligible".*

Some patients (P2 and P7) underestimated the severity of disease recurrence, harboured a sense of complacency, or experienced a rapid decline in motivation to take medication after symptoms subsided.

*"I always took chances, thinking I wouldn't relapse. After my stomach stopped hurting, I figured since it didn't hurt anymore, there was no need to take medicine every day".*

##### 4.1.3.3 Low patient self-efficacy

During the interviews, it was found that some patients (P9) exhibited excessive dependence on medical care and had insufficient self-management capabilities.

*"Over the past few years, I've been to major hospitals all over the country, but none of them could diagnose this illness. Now the doctor has me taking medication, but after taking it, my stomach hurts even worse. Then the doctor told me to get injections. I really don't know what to do anymore. Should I listen to the doctor? And which doctor should I listen to?"*

Additionally, the interviews revealed that medication adherence among some patients (P1, P2, and P15) was not integrated into stable daily routines. Many patients relied on active recall, with irregular schedules and busy lifestyles posing significant barriers. Changes in the patient’s physical environment—such as alterations to daily settings (e.g., business trips, travel)—frequently led to lapses in memory, making missed doses highly likely.

*"I rely entirely on my memory, and when I get busy, I forget. Sometimes after night shifts, I'm so groggy I can't even remember if I ate. I occasionally have to travel for work and stay in hotels. When my schedule gets thrown off, I completely forget to take my medication".*

## 5 Discussion

### 5.1 Deficiencies in the capability dimension influence patient behaviour

The capacity impairment identified in this study directly corresponds to the “capacity” dimension within the COM-B model. It manifests as patients’ inadequate understanding of the nature of their illness and medication principles and is associated with current doctor-patient communication patterns and systemic deficiencies in health education. This core deficiency manifests as inadequate knowledge reserves and a lack of self-management skills. This is consistent with research findings indicating that self-management capabilities for chronic conditions influence health behaviours (18). Effective self-management techniques and lifestyle modifications may play a crucial role in sustained self-care by empowering women with greater control over their health and providing valuable support for their existing treatment regimens. Given the prolonged disease course and frequent adverse drug reactions in patients with OE, the body experiences multiple symptoms after long-term medication. To effectively manage this “protracted battle” and adapt to new living conditions, patients require relevant knowledge and skills in self-management to enhance medication adherence and improve quality of life. This explains why functional impairments are particularly pronounced among patients with OE. The root cause of these impairments may lie in inadequate doctor-patient communication and inefficient health education formats.

#### 5.2 Multiple constraints within the opportunity dimension lead to patient non-compliance

From an opportunity perspective, this encompasses external factors such as the physical environment, socio-cultural context, and macro-level systems. Of paramount importance is the significant issue of financial burden, which places pressure on the majority of patients—particularly those without stable income, low medical insurance reimbursement rates, or no employee-based health insurance. This aligns with the findings of a 2024 meta-analysis (19). This study indicates that healthcare costs and out-of-pocket expenditures are among the most critical factors contributing to medication non-adherence in chronic disease management, particularly in low- and middle-income countries. The high out-of-pocket costs, through the mechanism of financial toxicity, force patients to make difficult choices between health and economic survival. This ultimately leads to treatment discontinuation, reduced dosages, or delayed medication use, worsening disease progression, and potentially increasing the overall healthcare burden on society due to complications (20). For a disease such as OE that requires decades of management, the financial burden is persistent and very real.

Additionally, studies have found that issues such as shortages of healthcare personnel and imbalances in the distribution of medical resources between urban and rural areas further limit patients’ access to high-quality medical resources and information (21, 22). This aligns with the findings of this study. Regarding social support, after long-term medication use, family members’ motivation for supervision diminishes, leading to a lack of positive reinforcement mechanisms. Patients experience a lack of continuous behavioural feedback and incentives, which increases their psychological fatigue.

Societal stigmatization of gynaecological conditions leads to intense feelings of shame among patients, presenting a key and unique barrier for OE as a gynaecological disorder. A 2023 study highlighted that unmarried young women face more severe gynaecological stigma and threats to their social identity (23). This aligns with the perspective presented in this study. Through the lens of social identification theory, patients internalize external stigma and begin defining themselves through these negative labels. This is not merely passive acceptance but an active—though often unconscious—psychological processing. To cope with anticipated discrimination and internal shame, patients frequently adopt coping strategies such as secrecy and concealment, selectively disclosing their condition in social and medical contexts. This ongoing identity management consumes significant psychological resources, intensifies mental stress, and may reduce medication adherence or even delay diagnosis and treatment, thereby creating a vicious cycle.

#### 5.3 Intrinsic barriers within the motivational dimension undermine patients’ motivation for treatment

The core “motivation” dimension within the COM-B model pertains to patients’ cognitive processes, affective evaluations, and decision-making. Barriers to this dimension stem from patients’ cognitive biases and healthcare providers’ habitual tendencies. At the patient level, excessive concern over hormonal side effects represents a classic challenge in chronic disease management. When patients’ fear of side effects outweighs their anxiety about disease recurrence, their adherence motivation plummets. Patients with OE worry about metabolic abnormalities, mood swings, thrombosis risks, and fertility impacts, leading to reduced medication adherence. All these factors weaken intervention motivation. Research indicates that high self-efficacy encourages individuals to attribute health management to factors within their control, thereby enhancing their initiative in goal setting, skill development, and behavioural change (24). Conversely, patients with OE commonly exhibit low self-efficacy and excessive reliance on medical interventions, making them prone to fatigue and motivational exhaustion during long-term treatment.

In summary, the value of this study lies in revealing through the COM-B model that dienogest adherence among patients with OE is not attributable to a single factor but rather stems from an interwoven complex behavioural system. Motivational barriers represent the ultimate outcomes of capability and opportunity barriers. Patients’ cognitive biases and fear of side effects, amplified by cumbersome follow-up procedures and diminished family support, ultimately lead to motivational exhaustion. This highlights the dynamic and mutually reinforcing relationships among the components of the COM-B model. Barriers across these three dimensions reinforce each other; therefore, any effective intervention strategy must adopt a systemic approach and implement multifaceted integrated interventions.

### 5.4 Implications for nursing practice

#### 5.4.1 Capability dimension: establishing educational and support systems

To address “capacity” barriers, a structured, individualised empowerment education system must be established. In health education: ① Employ visual aids (e.g., pharmacokinetic diagrams, animated demonstrations) to explain drug mechanisms in accessible terms, clarifying immediate and long-term consequences of missed doses; ② Nurses, as core medication management practitioners and interventionists, should lead personalised education programme design, enhancing their role in improving patient medication adherence (25). Establish a nurse-led intervention framework with standardised guidelines for missed-dose management, reinforced through pocket guides to aid recall of catch-up dosing protocols. Implement rapid adverse reaction consultation channels (e.g., via dedicated apps or mini-programmes) to provide tiered management advice, thereby reducing irrational discontinuation driven by patient anxiety.

Enhancing doctor-patient communication and education is also paramount. Clinicians should provide thorough and clear patient explanations regarding the condition and medication, manage patient expectations, detail potential side effects and their management, address missed doses, assess and address patient concerns, and collaboratively develop personalised treatment plans. Effective side effect management should be offered, including explanations, reassurance, and potential solutions for issues such as irregular bleeding, alongside proactive management of other quality-of-life-impacting side effects. Additionally, establishing disease support groups for patients with OE, organising regular health lectures, and providing platforms for learning and interaction can ensure service accessibility. Implementing structured follow-up schedules, proactively enquiring about adherence and side effects, and utilising these appointments for re-education are recommended. Consider employing SMS or WeChat platforms to remind patients of appointments and medication schedules.

#### 5.4.2 Opportunity dimension: fostering supportive care environments and policy safeguards

At the opportunity dimension level, efforts should be made to foster supportive care environments. Enhancing medication adherence among patients with OE requires collaborative contributions from families, communities, hospitals, and broader societal sectors. Single-source support models often prove insufficient to facilitate optimal therapeutic outcomes, underscoring the need for robust external support systems. In this process, clinical electronic health records can be leveraged to construct patient health profiles, develop predictive models for OE, and establish dedicated multi-stakeholder information exchange platforms tailored to individualised disease management requirements (26). Community hospitals, as primary healthcare touchpoints, must address the last-mile challenge by establishing mechanisms to prevent medication discontinuation. Family members should be actively engaged, with emphasis placed on understanding patients’ psychological states and practical needs, enabling them to effectively fulfil dual roles of supervisory support and daily assistance.

In terms of policy, efforts should be made to explicitly include ovarian endometriosis within the scope of outpatient special chronic disease coverage under medical insurance. This would permit patients to obtain regular prescriptions during outpatient visits while benefiting from reimbursement rates comparable to those of inpatient care. Furthermore, dydrogesterone should be prioritised for inclusion in the National Essential Medicines List, with provisions for dynamic adjustment and updating. Furthermore, while inclusion is fundamental, the critical factor lies in establishing a high reimbursement rate to significantly reduce out-of-pocket expenses per prescription. It is essential to ensure that reimbursement covers the entire treatment cycle, including preoperative suppression, postoperative relapse prevention, and long-term medication management. This is crucial for patients who require sustained treatment.

#### 5.4.3 Motivational dimension: implementing motivational interventions centred on enhancing self-efficacy

At the motivational level, motivational interventions centred on enhancing self-efficacy should be implemented. Addressing patients’ cognitive biases requires reinforcing personalised therapeutic feedback and assisting them in setting a series of phased, concrete, and achievable short-term goals. Through visualised medication effects—such as regular cyst ultrasound examinations, pain score records, and medication diaries—successful experiences accumulate to establish a positive feedback mechanism. Guide patients to summarise their personalised management strategies and build tangible achievements that demonstrate treatment efficacy. Establishing peer support groups via online WeChat communities or offline gatherings/seminars to encourage medication experience sharing. Peer-to-peer empathetic support alleviates stigma, reassuring patients they are not alone while reinforcing the belief ‘I can do this’ through successful peer examples. For emotional management, we collaborated with the psychology department to establish a missed-dose emotional support mechanism. Guide patients via psychological helplines or online modules to accept occasional missed doses and avoid excessive self-blame. For patients experiencing infertility anxiety with thoughts such as ‘I worry I will not conceive’, develop tailored health education with the reproductive medicine team. Inform patients that ‘the vast majority achieve natural conception through medication or surgical treatment’, and that ‘reproductive assistance programmes like IVF may also fulfil their desire to become mothers’.

For healthcare professionals, training is required to shift the perception of ‘prioritising surgical interventions’. Standardised communication manuals and personalised follow-up tools should be developed to enhance attention to patients’ lifestyle factors and financial burdens. Nurse-led training courses focusing on medication adherence motivation should be introduced to improve proactive interventions. Long-term patients should be offered incentives, such as priority appointments, to reinforce positive experiences with consistent medication use. Patients should be encouraged to raise queries and discuss treatment plans jointly, thereby strengthening their active engagement in the therapeutic process.

## 6 Summary

This study employed semi-structured interviews in conjunction with the COM-B behavioural change model to systematically analyse barriers to dienogest medication adherence among patients with ovarian endometriosis. It explored the experiences and perceptions of patients with OE during medication use in depth. Based on the interview findings, specific nursing practice implications were proposed, providing a reference for subsequent intervention strategy development to enhance patient medication adherence.

## 7 Research limitations and future directions

This study has the following limitations. First, the representativeness of the sample is constrained, as the data originates from 15 patients at a single centre in China, potentially failing to represent patient groups attending community hospitals or primary healthcare facilities. The participants predominantly comprised married, childbearing middle-aged and young adults, lacking coverage of adolescents, childless individuals with high anxiety, and low-income rural populations, thereby potentially lacking generalisability. Second, at the methodological level, qualitative interviews are susceptible to social desirability bias. Social desirability bias, a common limitation in qualitative research, refers to respondents’ tendency to provide answers conforming to social norms or researcher expectations rather than entirely truthful feedback. To mitigate this bias, we employed neutral, non-leading questioning in our research design and fostered a secure, confidential environment during interviews, encouraging participants to share genuine experiences. Nevertheless, patients may still inadvertently exaggerate medication adherence, downplay concerns about medications, or embellish family support, thereby affecting the authenticity and depth of the revealed impediments. Finally, this study did not analyse socio-cultural factors such as the associations between educational attainment, cognitive biases, and barriers to adherence, which may significantly influence cognitive distortions and health literacy. For instance, patients from differing educational backgrounds may exhibit disparities in their capacity to comprehend complex medication regimens or access reliable health information, potentially impacting their self-management abilities and motivation to adhere. Future research could incorporate these sociodemographic variables as moderating or analytical factors to gain deeper insights into the variability in disease distribution, thereby enabling the development of more stratified and targeted intervention strategies.

In summary, future clinical practice can build upon these research findings to develop nurse-led intervention programmes grounded in theoretical frameworks, such as self-efficacy theory, tailored to the physiological characteristics and personalised needs of patients with OE. This should be pursued through large-scale, multicentre, collaborative, rigorously designed randomised controlled trials (RCTs).

## Data Availability

Data are available from the corresponding author on reasonable request.
