# Supplementary material for: Barriers affecting medication adherence to dienogest among patients with ovarian endometriosis: a qualitative study
Source: Front Med (Lausanne). 2026 Jan 30;13:1749740. doi: 10.3389/fmed.2026.1749740 (PMC12901324; doi:10.3389/fmed.2026.1749740)
Supplement: Supplementary file 1 [file Table_1.docx]

**Supplementary Material: Alignment of Interview Guide with the COM-B Model**

| **Interview questions** | **dimension** | **Note** |
| --- | --- | --- |
| 1.Has your doctor explained the mechanism of action of dienogest and why it must be taken long-term? | **Capability, C** | Assess the patient's level of understanding regarding the mechanism of action of the medication and the necessity of long-term treatment. |
| 2.Are you clear on the rules for taking a missed dose? | **Capability, C** | Assess whether the patient possesses the knowledge to correctly manage missed doses. |
| 3.Do you understand how to manage common side effects? | **Capability, C** | Assess patients' knowledge and coping abilities regarding the management of drug side effects. |
| 4.What measures do you take after missing a dose? | **Capability, C** | Further examine patients' behavioural responses and knowledge application in real-world scenarios. |
| 5.Does the cost of dienogest cause financial strain? How much is covered by insurance? | **Opportunity, O** | Assess the extent to which external economic resources and medical insurance policies support patients' access to medication. |
| 6.Does the follow-up appointment process affect your confidence in continuing medication? | **Opportunity, O** | Assess the convenience and level of support provided by the healthcare system. |
| 7.Are your family members aware of your medication needs? Do they help remind or monitor your medication? | **Opportunity, O** | Assess the effectiveness of family and social support systems. |
| 8.Does your work/study environment facilitate taking medication on time? | **Opportunity, O** | Assess the convenience and degree of disruption to medication-taking behaviour within the daily environment. |
| 9.What factors have motivated you to adhere to medication? What emotions arise when you miss a dose? Do these emotions affect subsequent medication adherence? | **Motivation, M** | Assess the patient's intrinsic motivation, emotional responses, and their impact on behaviour. |
| 10.Please describe a typical scenario where you missed a dose. | **Comprehensive（Capability, C + Opportunity, O + Motivation）** | Through specific contextual reviews, comprehensively examine how three categories of factors collectively influence behaviour. |

Note: This table illustrates the alignment between the semi-structured interview guide used in this study and the COM-B model of behavior change. Except for the tenth question, each interview question was designed to target one dimensions of the model (Capability, Opportunity, Motivation), aiming to systematically explore the barriers affecting dienogest medication adherence in patients with ovarian endometriosis.
